# Developing attention deficits/hyperactivity disorder-virtual reality diagnostic tool with machine learning for children and adolescents

**DOI:** 10.3389/fpsyt.2022.984481

**Published:** 2022-09-21

**Authors:** Tjhin Wiguna, Raymond Bahana, Bayu Dirgantoro, Kusuma Minayati, Sylvie Dominic Teh, Raden Irawati Ismail, Fransiska Kaligis, Ngurah Agung Wigantara

**Affiliations:** ^1^Faculty of Medicine, Universitas Indonesia – Dr. Cipto Mangunkusumo General Hospital, Jakarta, Indonesia; ^2^Faculty of Computer Science, Bina Nusantara University, Jakarta, Indonesia; ^3^Dr. Cipto Mangunkusumo General Hospital, Jakarta, Indonesia

**Keywords:** ADHD, virtual reality, diagnostic tool, Indonesia, machine learning

## Abstract

The traditional diagnosis of Attention Deficits/Hyperactivity Disorder (ADHD) is through parent-child interviews and observations; therefore, innovative ADHD diagnostic tools that represent this digital era are needed. Virtual reality (VR) is a significant technology that can present a virtual immersive environment; it can provide an illusion of participation in an artificial milieu for children with ADHD. This study aimed to develop an ADHD-VR diagnostic tool construct (Research Domain Construct/RDC) based on the DSM5 ADHD diagnostic criteria, and using the RDC to develop a diagnostic tool with a machine learning (ML) application that can produce an intelligent model to receive some complex and multifaceted clinical data (ADHD clinical symptoms). We aimed to expand a model algorithm from the data, and finally make predictions by providing new data (output data) that have more accurate diagnostic value. This was an exploratory qualitative study and consisted of two stages. The first stage of the study applied the Delphi technique, and the goal was to translate ADHD symptoms based on DSM 5 diagnostic criteria into concrete behavior that can be observed among children in a classroom setting. This stage aimed to gather information, perceptions, consensus, and confirmation from experts. In this study, three rounds of Delphi were conducted. The second stage was to finalize the RDC of the ADHD-VR diagnostic tool with ML, based on the first-stage results. The results were transformed into concrete activities that could be applied in the programming of the ADHD-VR diagnostic tool, followed by starting to input data that were required to build the diagnostic tool. The second stage consisted of more than ten focus-group discussions (FGDs) before it could be transformed into the ADHD-VR diagnostic tool with the ML prototype. First-stage data analysis was performed using Microsoft Excel for Mac. Qualitative data were analyzed using conceptual content analysis with a manifest/latent analysis approach. From the first stage of the study, there were 13 examples of student behaviors that received more than 75% totally agreed or agreed from the experts. The RDC of the ADHD-VR diagnostic tool with machine learning application consisted of three domains and was divided into six sub-domains: reward-related processing, emotional lability, inhibitory, sustained attention, specific timing of playing in order, and arousal. In conclusion, the results of this study can be used as a reference for future studies in a similar context and content, that is, the ADHD-VR diagnostic tool with machine learning based on the constructed RDC.

## Introduction

The Fourth Industrial Revolution (FIR) and digital revolution era have grown significantly over the last 10 years. The characteristics of this meaningful period are shown by disruptive technologies that can associate with the physical, digital, and biological aspects of the environment, for example, in health systems and services. The expanded usage of 4.0 technologies is expected to increase the precision, efficiency, and effectiveness of medical diagnostic tools, treatment approaches, and health monitoring to anticipate the influence of many diseases (1). Therefore, based on the extension of the FIR definition in the health industry, new concepts of digital health systems are being transformed, for example, in personalizing diagnosis or treatment for specific diseases to promote better outcomes.

Virtual reality (VR) is one of the most significant and innovative tools that encompasses FIR technologies in medical practices, especially in child and adolescent psychiatry in which (i) diagnostic approaches mainly depend on parents’ report questionnaires, psychiatric interviews, and observations; and (ii) treatment consideration has been limited to interpersonal psychotherapy and medication. Based on several studies, VR is defined as real-time interactive graphics with 3D or 4D models that construct the objective of a virtual immersive environment, thus giving an illusion of participation in an artificial milieu rather than a real external observation of such a situation (2). The VR component consists of three- and four-dimensional models of the environment with stereoscopic head-tracker displays, hand/body tracking, and binaural sounds that can provide immersive, multi-sensory experiences for the users (3, 4).

Attention Deficits/Hyperactivity Disorder (ADHD) is a prominent mental health problem, especially among primary school children, and its worldwide prevalence is estimated to be 5 – 13% (5). The main symptoms are categorized into two domains: inattentive symptoms and hyperactivity-impulsivity symptoms that have persisted for at least 6 months. The other characteristics are symptoms that can be observed before the age of 12 years and are not consistent with the developmental milestones of children of similar age; moreover, it significantly impacts social and academic activity. Consequently, children with ADHD have several difficulties in daily functioning, such as academic achievement, social life, and learning capabilities, which may reduce their quality of life (6, 7). Therefore, early detection and precise diagnosis are crucial. In traditional child and adolescent psychiatry services, psychiatric interviews, observations, and the use of parents/teacher-rating questionnaires are the foremost approaches to ADHD diagnostic procedures (2). Therefore, subjective perception may influence the decision during diagnosis, especially for ambiguous cases that look like ADHD, such as borderline intellectual functioning, childhood anxiety and depression, and learning disorders. Therefore, diagnostic tools are important in ADHD cases, as they may provide information and evidence that confirms diagnosis, provides differential diagnosis, and gives parents more factual data according to the ADHD diagnosis and further treatment approach. Although biological marker research on ADHD is currently very developed, the cost of such examinations is very high, and few families can afford it. Meanwhile, there are several tools or examinations that can serve as an adjunct diagnostic approach for ADHD; however, it is not specific enough to identify the clinical symptoms of ADHD. They are mostly developed to identify attention or cognitive problems, and several devices have been used to elaborate on the neuropsychology and electrophysiology of the brain (8–11).

Consequently, developing an ADHD diagnostic tool based on ADHD clinical symptoms is important because it may complete and validate ADHD diagnosis based on the traditional approach (2). Several studies have shown that VR can be applied as a diagnostic tool for ADHD because of its ability to create a virtual environment that is free of distress, entertaining, and it can gather and analyze better behavioral, inattention, and distractibility symptoms than ordinary neuropsychological tests (2, 12, 13). Recently, continuous performance tests (CPT) and other neuropsychological tests have been used as standardized tests, even if they may not be very specific for ADHD diagnosis but more focused on attentional problems (14–16). Moreover, several ADHD-VR diagnostic tools that have been developed, such as the Virtual Reality Medical Center system (VRMC, China) and AULA VR-based test (Spain) followed the constructs of CPT in assessing ADHD symptoms/diagnosis. VRMC showed a beneficial effect in differentiating between children with ADHD and learning problems; however, it mainly reflected visual attention (17). Meanwhile, AULA appeared to be effective in differentiating children with and without attentional problems, which can provide additional information for ADHD diagnosis (18). An overview study by Goharinejad et al. (19) revealed that among 30 studies that were reviewed during 2010–2022, only seven operated VR that focused on improving ADHD diagnosis, ten utilized VR in assessing ADHD symptoms, and only one study incorporated a machine learning (ML) technology to improve diagnosis accuracy. The overview study results showed that VR technologies were especially useful in improving the evaluation of ADHD symptoms and increasing the accuracy of ADHD diagnosis in children.

Moreover, machine learning (ML) applications produce an intelligent model that can receive complex and multifaceted clinical data (ADHD clinical symptoms), expand a model algorithm from the data, and finally make predictions by providing new data (output data) that have more accurate diagnostic value (20). Therefore, it may improve the accuracy of the ADHD VR diagnostic tool during the assessment process (2, 21, 22). For example, Yeh et al. (21) developed an immersive VR classroom system with intelligent assessment to assist in ADHD diagnosis. The activities embedded in the VR classroom system consisted of sustained and selective attention tasks with visual, auditory, and visual-auditory distractors during task completion. In addition, a machine-learning application can incorporate task performance and neurobehavioral sensing to differentiate between ADHD and non-ADHD cases. The study showed that the mean accuracy for repeated cross-validation was 83.2%, and it was concluded to be a good system to assist clinicians in ADHD diagnosis. Thus, ADHD-VR diagnostic tool with ML application can be said as useful equipment in the near future, but most studies did not describe fully the development processes of those ADHD-VR diagnostic tools and research domain construct, respectively.

Therefore, this study was designed to build an ADHD-VR diagnostic tool construct (Research Domain Construct/RDC) based on the DSM5 ADHD diagnostic criteria, and to use the RDC to develop an ADHD-VR diagnostic tool with a ML model. RDC may explain the correlation between several domains of ADHD symptoms and basic engineering principles to develop a dynamic software construction into ADHD-VR diagnostic tool platforms (23, 24). Based on Baroni and Castellanos (25) and Benyakorn et al. (26), an optimal ADHD diagnostic tool construct consists of at least three components and six subcomponents; (i) immediate feedback is relevant and occurs as close as possible in time to when the behavior is produced (sustained attention and inhibition that consist of ADHD symptomatology, task to be completed, distractors, and more); (ii) schedule setting covers goal and time setting to address condition with task completion and time management (emotion lability and reward-related processing that are generated into specific goals, task completion, game mechanics, and more); and (iii) difficulty matching incorporates adapting to the child’s current level of functioning and may change as the child’s behavior changes (arousal and timing that can be specified into time management, the current level of child adaptation style, what should be observed to assist the ADHD diagnosis, and more) (27, 28).

## Methods

The study design followed the first and second steps of the four-step method of ADHD VR diagnostic tool development (2). This study consisted of two stages. The first and second stages were designed as exploratory qualitative research to build the RDC and ADHD-VR diagnostic tool with an ML prototype (24, 29). The research protocol was approved by the Ethics Committee of the Faculty of Medicine, University of Indonesia, Jakarta, Indonesia (reference number: KET-583/UN2.F1/ETIK/PPM.00.02/2019).

The first stage of the study applied the Delphi technique, and the goal was to translate ADHD symptoms based on the DSM 5 diagnostic criteria into concrete behavior that can be observed among children in a classroom setting. This stage aimed to gather information, perceptions, consensus, and confirmation from experts. There were three rounds of Delphi and involved seven experts consisting of: (ii) three child psychiatrists, (ii) two child psychologists, and (iii) two school counselors who also acted as primary school teachers. The first and second rounds were designed as qualitative assessments to gather information and perception about the DSM 5 ADHD diagnostic criteria that can be observed among primary school student behaviors in classroom settings. The third round was designed as a quantitative assessment to obtain consensus or confirmation among experts according to the second-round results. Because of the coronavirus disease 2019 (COVID-19) pandemic, each round of the Delphi method was completed by sending the questionnaire through the participants’ email. The results of each round were sent to all the participants using questionnaires until a consensus was reached.

The second stage was to finalize the RDC of the ADHD-VR diagnostic tool with ML, and followed by developing the ADHD-VR diagnostic tool prototype. Through the FGDs on the second stage, the consensus from the first-stage was transformed into RDC based on Baroni and Castellanos (25) and Benyakorn et al. (26) theoretical concept. Following the RDC, FGDs continued to explore input into concrete activities that could be applied in the programming of the ADHD-VR diagnostic tool prototype. The second stage consisted of more than ten focus-group discussions before it could be transformed into the ADHD-VR diagnostic tool prototype. Furthermore, FGDs addressed several frameworks that translated the RDC into ADHD-VR-based game coding, including transforming the input and output data into a single VR-based game platform that could assess ADHD clinical symptoms. During the second stage, FGDs discussed (i) the VR game concept, strategies, visual appearances, characters and player interactions, and duration of VR playing (minutes) that could assess ADHD clinical symptoms; (ii) game design, including genre, game mechanics (game data and game engine; the Unity game engine was used in the ADHD-VR diagnostic tool development), play mechanics, and play experiences. At this stage, the game designers also discussed the visual and auditory themes, characters, classroom environment, storyboard, and distractors (both visual and auditory); and (iii) the technical game analysis, which included the programming technique of the ADHD-VR diagnostic tool framework and software, including the selection of the game engine, ML application, data management, ADHD-VR diagnostic tool performance, and optimization. Each FGD event was recorded and transcribed. FGDs included a child psychiatrist and general psychiatrist from the Child and Adolescent Psychiatry Division, Department of Psychiatry, Faculty of Medicine, Universitas Indonesia - dr. Cipto Mangunkusumo General Hospital, Jakarta, Indonesia; and a computer scientist and game designer specialist from the Faculty of Computer Science of Bina Nusantara International University, Jakarta, Indonesia.

### Questionnaire

In the first stage of the study, a specific questionnaire was developed to build the RDC of the ADHD-VR diagnostic tool. In the first round, the study provided a questionnaire that could facilitate experts to express their perspectives and opinions on primary school student activities that may be observed in classroom settings based on the DSM 5 ADHD diagnostic criteria for inattentive, hyperactivity, and impulsivity symptoms. The questionnaire consisted of open-ended questions that asked, “Can you describe these symptoms based on your observation of the attitude and behavior of your patients/students with ADHD in the classroom setting?” The second-round questionnaire was developed based on the first-round results and performed as a table of statements. In the second round, experts answered the statements scored on a Likert scale: totally agree – 4, agree – 3, somewhat agree – 2, and disagree – 1. In addition, the second-round questionnaire was also included with comment columns so that experts could provide their feedback and judgments on the statements, if necessary. The last round questionnaire was built from the second-round results, inputs were modified, experts’ comments were followed, and statements that were rated as somewhat agree or disagree were excluded. In the third round, experts were invited to answer the statements on a Likert scale once more, and it was categorized as totally agree – 4, agree – 3, somewhat agree – 2, and disagree – 1. The final round was the consensus and confirmation phase. The level of consensus used in this study was 75% of agree or totally agree statements.

### Data analysis

**FIGURE 1:**
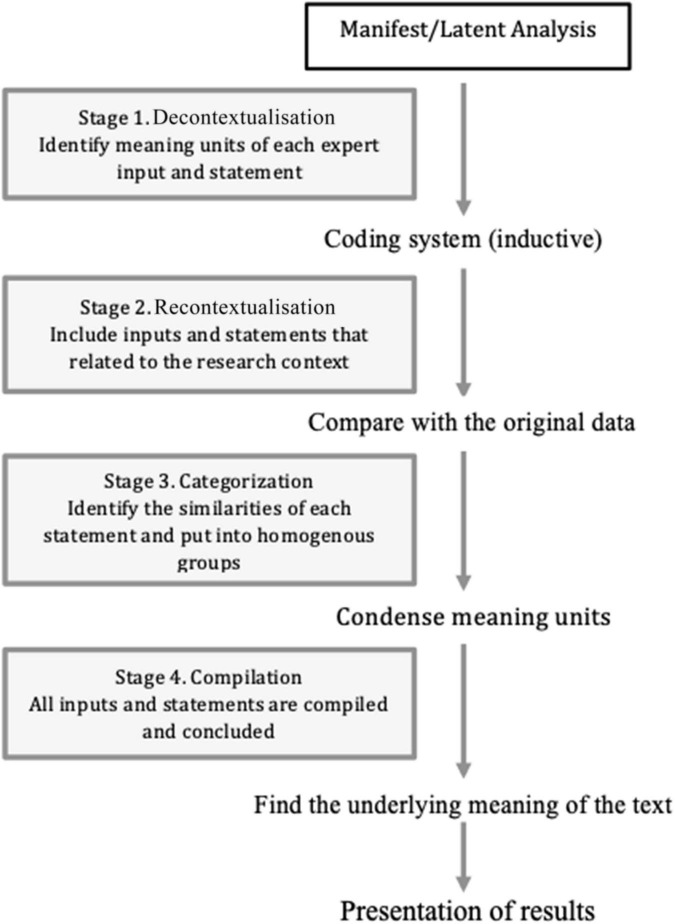
Manifest/latent qualitative data analysis based on Bengtsson (30), p. 9.

In the first stage, the data analysis was performed using Microsoft Excel for Mac. Qualitative data were analyzed using conceptual content analysis with a manifest/latent analysis approach that consisted of four steps: decontextualization, recontextualization, categorization, and compilation (Figure 1). The consensus on the third round of the first stage was defined as minimum of 75% agree and totally agree statements. The second stage, FGDs was recorded and transcribed verbatim using Microsoft Word. The research domain construct of the ADHD-VR diagnostic tool prototype was created using an inductive method based on conceptual content analysis (30).

## Results

The expert consensus is shown in Table 1.

This study aimed to develop an ADHD-VR diagnostic tool using ML, which consists of three stages, including feasibility testing. The conceptual content analysis from the first and second stages resulted in the ADHD-VR diagnostic tool prototype with an ML construct and general agreement with the ADHD-VR diagnostic tool prototype.

**TABLE 1:** Expert consensus on attention deficits/hyperactivity disorder (ADHD) classroom behavior based on DSM-5 and RDC.

No.	ADHD symptoms based on DSM 5 ADHD diagnostic criteria	Observed behavior	Consensus rate	RDC
1	Often fails to give close attention to details or makes careless mistakes in schoolwork, at work, or during other activities (e.g., overlooks or misses details, work is inaccurate).	1. Number of tasks completed correctly in a specified time/during tasks completion/examination (according to given verbal and auditory stimuli/instructions).	92%	Reward-related processing, Emotional lability, Inhibitory, Sustained attention, Specific timing of playing in order, Arousal
		2. Number of mistakes made during tasks completion/examination (not according to given verbal and auditory stimuli/instructions).	92%	
2	Often has difficulty sustaining attention in tasks or play activities (e.g., has difficulty remaining focused during lectures, conversations, or lengthy reading).	1. Duration needed to complete each task given in a specified time/during tasks completion/examination (measured as starting when the instruction is given until the task is completed) ->summed up at the end of the examination.	83%	
		2. Number of mistakes made during tasks completion/examination (not according to given verbal and auditory stimuli/instructions).	92%	
3	Often does not seem to listen when spoken to directly (e.g., mind seems elsewhere, even in the absence of any obvious distraction).	1. Number of mistakes made during tasks completion/examination (not according to given verbal and auditory stimuli/instructions).	83%	
		2. Number of tasks completed correctly in a specified time/during tasks completion/examination (according to given verbal and auditory stimuli/instructions).	83%	
4	Often does not follow through on instructions and fails to finish schoolwork, chores, or duties in the workplace (e.g., starts tasks but quickly loses focus and is easily sidetracked).	1. Number of mistakes made during tasks completion/examination (not according to given verbal and auditory stimuli/instructions).	73%	
		2. Time needed to complete each task given in a specified time/during tasks completion/examination (measured as starting when the instruction is given until the task is completed) ->summed up at the end of the examination.	82%	
5	Often has difficulty organizing tasks and activities (e.g., difficulty managing sequential tasks; difficulty keeping materials and belongings in order; messy, disorganized work; has poor time management; fails to meet deadlines).	1. Number of mistakes made when completing tasks regarding the sequence of the verbal and auditory stimuli/instructions.	83%	
		2. Number of verbal and auditory stimuli/instructions sequence completed in the correct order.	92%	
		3. Number of tasks completed correctly during tasks completion/examination (according to given verbal and auditory stimuli/instructions).	75%	
6	Often avoids, dislikes, or is reluctant to engage in tasks that require sustained mental effort (e.g., schoolwork or homework; for older adolescents and adults, preparing reports, completing forms, reviewing lengthy papers).	1. Number of tasks completed correctly in a specified time/during tasks completion/examination (according to given verbal and auditory stimuli/instructions).	83%	
		2. Number of verbal and auditory stimuli/instructions sequence completed in the correct order.	75%	
		3. Number of mistakes made when completing tasks regarding the sequence order of the verbal and auditory stimuli/instructions.	58%	
7	Often loses things necessary for tasks or activities (e.g., school materials, pencils, books, tools, wallets, keys, paperwork, eyeglasses, mobile telephones).	1. Number of tasks completed correctly in a specified time/during tasks completion/examination (according to given verbal and auditory stimuli/instructions).	42%	
		2. Number of mistakes made during tasks completion/examination (not according to given verbal and auditory stimuli/instructions).	42%	
8	Often easily distracted by extraneous stimuli (for older adolescents and adults, may include unrelated thoughts).	1. Number of tasks completed correctly in a specified time/during tasks completion/examination (according to given verbal and auditory stimuli/instructions).	100%	
		2. Number of mistakes made during examination (not according to given verbal and auditory stimuli/instructions).	92%	
9	Often forgetful in daily activities (e.g., doing chores, running errands; for older adolescents and adults, returning calls, paying bills, keeping appointments).	1. Number of tasks completed correctly in a specified time/during tasks completion/examination (according to given verbal and auditory stimuli/instructions).	75%	
		2. Number of mistakes made during tasks completion/examination (not according to given verbal and auditory stimuli/instructions).	92%	
10	Often fidgets with or taps hands or feet or squirms in seat.	1. Amount of movement marked from the start of executing given instructions (since the verbal and auditory stimuli/instructions are given until they are completed, and the times between).	92%	
		2. Time needed to complete the whole set of verbal and auditory stimuli/instructions given.	83%	
		3. Movement pattern of each child.	75%	
11	Often leaves seat in situations when remaining seated is expected (e.g., leaves his or her place in the classroom, in the office or other workplace, or in other situations that require remaining in place).	1. Amount of movement marked from the start of executing given instructions (since the verbal and auditory stimuli/instructions are given until they are completed, and the times between).	83%	
		2. Time needed to complete the whole set of verbal and auditory stimuli/instructions given.	83%	
		3. Movement pattern of each child.	73%	
12	Often runs about or climbs in situations where it is inappropriate. (Note: In adolescents or adults, may be limited to feeling restless.)	1. Amount of movement marked from the start of executing given instructions (since the verbal and auditory stimuli/instructions are given until they are completed, and the times between).	92%	
		2. Time needed to complete the whole set of verbal and auditory stimuli/instructions given.	92%	
		3. Movement pattern of each child.	82%	
13	Often “on the go,” acting as if “driven by a motor” (e.g., is unable to be or uncomfortable being still for extended time, such as in restaurants, meetings; may be perceived by others as being restless or difficult to keep up with).	1. Amount of movement marked from the start of executing given instructions (since the verbal and auditory stimuli/instructions are given until they are completed, and the times between).	83%	
		2. Time needed to complete the whole set of verbal and auditory stimuli/instructions given.	73%	
		3. Movement pattern of each child.	73%	
14	Often unable to play or engage in leisure activities quietly.	1. Observation during examination with VR technology: can the child stay quiet or not	83%	
		2. Amount of movement marked from the start of executing given instructions (since the verbal and auditory stimuli/instructions are given until they are completed, and the times between).	75%	
15	Often talks excessively.	1. How many tasks has the patient started working on before the verbal and auditory instructions/stimuli were given completely?	67%	
		2. How long does it take since the verbal and auditory stimuli/instructions were given till the patient starts working on it?	92%	
16	Often blurts out an answer before a question has been completed (e.g., completes people’s sentences, cannot wait for turn in conversation).	1. How many tasks has the patient started working on before the verbal and auditory instructions/stimuli were given completely?	92%	
		2. How long does it take since the verbal and auditory stimuli/instructions were given till the patient starts working on it?	92%	
17	Often has difficulty waiting his or her turn (e.g., while waiting in line).	1. How many tasks has the patient started working on before the verbal and auditory instructions/stimuli were given completely?	58%	
		2. How long does it take since the verbal and auditory stimuli/instructions were given till the patient starts working on it?	50%	
18	Often interrupts or intrudes on others (e.g., butts into conversations, games, or activities; may start using other people’s things without asking or receiving permission; for adolescents and adults, may intrude into or take over what others are doing).	1. How many tasks has the patient started working on before the verbal and auditory instructions/stimuli were given completely?	92%	
		2. How long does it take since the verbal and auditory stimuli/instructions were given till the patient starts working on it?	91%	
		3. How many tasks are done in sequence order different from the given verbal and auditory instructions/stimuli?	70%	

### The first-stage results

From the first and second rounds of Delphi, the experts identified more than 50 examples of students with ADHD behavior in classroom settings based on 18 items of the DSM 5 ADHD diagnostic criteria. However, in the third round of Delphi, expert consensus showed only 13 examples of student behavior that could be represented by the DSM 5 diagnostic criteria for ADHD with 75% totally agree or agree statements (Table 1).

### The second-stage results

The focus group discussions during the second stage were based on the first-stage results. The FGDs results were generated by content conceptual analysis that consisted of decontextualization (Identify meaning units of each expert input and statement), recontextualization (Include inputs and statements that related to the research content), categorization (Identify the similarities of each statement and put into homogenous groups), and compilation (All inputs and statements compiled and concluded) to finalize the RDC of the ADHD-VR diagnostic tool. Based on the RDC and other compiled inputs, it was transformed into concrete activities that applied in the programming of the ADHD-VR diagnostic tool prototype with ML application.

#### The research domain construct of the attention deficits/hyperactivity disorder-virtual reality diagnostic tool prototype

**FIGURE 2:**
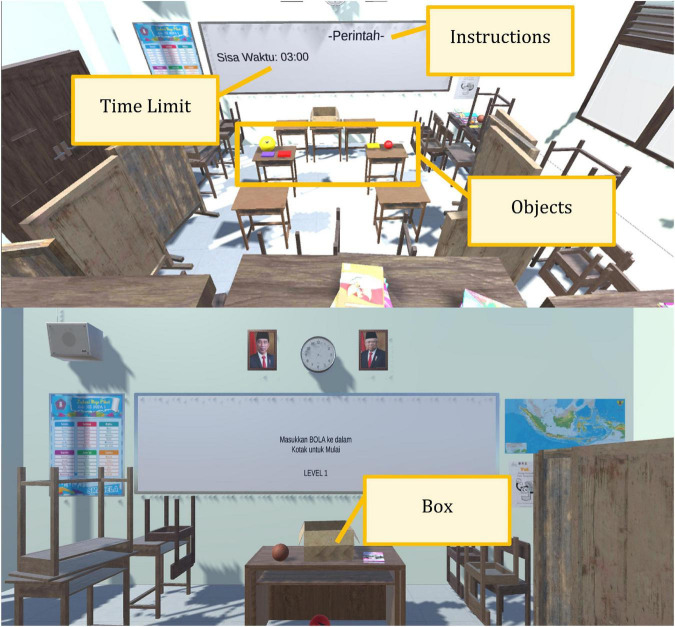
The attention deficits/hyperactivity disorder-virtual reality (ADHD-VR) diagnostic tool prototype appearances.

Schedule setting component, consisted of the following:

a. Reward-related processing aims to identify inattentive and hyperactivity-impulsivity symptoms. This subcomponent may be related to alterations in the dopaminergic and serotonergic systems of the ADHD brain. Therefore, activity is designed to examine the release of such neurotransmitters, such as dopamine and serotonin. Hence, a role-playing activity with challenging tasks was chosen (acting as a student in the classroom). The participants were instructed to complete several challenging tasks using visual and auditory instructions (to put specific objects that are defined as books and balls with random sizes and colors into the correct box; meanwhile, there are also several distractors that are defined as door opening sounds, sounds of children playing outside the classroom, sounds of animals, classroom tables, and chairs, including time limitation) (Figure 2). Every time an object is correctly placed in the box, the tool makes a nice sound. It is the only reward they receive, and there are no other rewards during examinations to avoid the tendency to focus on immediate rewards, which may lead to examination bias.

b. Emotional lability subcomponents are meant to quantify hyperactivity-impulsivity symptoms, especially those associated with emotion-related symptoms such as restlessness, difficulty in engaging in leisure activities, and difficulty in waiting turns. Thus, the challenging tasks are designed in several ways, such as objects (different colors and size books and balls) simultaneously appearing, using two types of instruction (auditory and visual), time limitation, and only using a nice sound but not other rewards as signs that students correctly or incorrectly completed their tasks. In addition, they cannot interact *via* chatting or personal data exchange, no data showing the points that were collected during training, and no data related to playing time, and more.

Immediate feedback component, consisted of the following:

a. The inhibitory subcomponent is arranged to identify inattentive, hyperactivity-impulsivity symptoms, and working memory. This arrangement is closely related to self-monitoring skills and goal-directed behaviors related to impulsive and hyperactive symptoms in children and adolescents with ADHD (Figure 2). The ADHD-VR diagnostic tool prototype had core mechanical qualities and followed the “Go/No-go” tasks combined with and without distractors; and it was done using the point-to-point game concept, specific timing, simultaneous order, and matching technique that may help to quantify hyperactivity-impulsivity symptoms.
b. The sustained attention subcomponents include organizational skills and working memory. The ADHD-VR diagnostic tool construct used a digital technology that tracked children and adolescent sustained attention skills by recording their ability to correctly memorize the specific tasks, when it was coupled with both auditory and visual distractors (various sounds, tables, and chairs in the classroom) that appeared simultaneously in real-time. Before and during task completion, participants need to sustain their attention, memorize the instructions (auditory or visual), and choose the right objects that appear concurrently. Meanwhile, they also need to organize their own strategies to complete their tasks. Thus, this immersion environment that is delivered through VR screens may quantify the participants’ ability to be selectively attentive to task completion, and also to organize their behavior in order to follow the given instruction (Figure 2). The software provided continuous monitoring of instructions and distractor appearances, along with tasks that needed to be accomplished.

Difficulty matching component, consisted of the following:

a. Specific timing of playing to identify the participant’s abilities to control and organize their behavior. The ADHD-VR diagnostic tool prototype was designed to recognize participant time management skills associated with inattention, hyperactivity-impulsivity, self-organization skills, and inhibition symptoms. Therefore, the total duration of the ADHD-VR diagnostic tool was designed for approximately 30 min of playing time, including a tutorial part. The faster they correctly completed the instructions, the better their motor timing, perceptual timing, and temporal foresight skills. Meanwhile, it also showed better attention span and self-organization skills.
b. The arousal subcomponents were designed to identify the participant’s ability to control distractions during task accomplishment. Once the participants correctly complete a task, the tool makes a nice sound that may or may not keep them aroused during the examination. In addition, it may or may not trigger the initiation skills. Therefore, this approach may also quantify inattentive and hyperactivity-impulsivity symptoms.

#### Developing and programming attention deficits/hyperactivity disorder-virtual reality diagnostic tool prototype with machine learning

**FIGURE 3:**
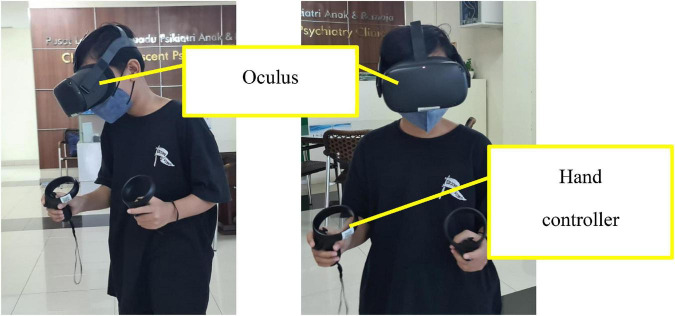
Attention deficits/hyperactivity disorder-virtual reality diagnostic tool.

The final compilation of the experts’ inputs during FGDs and based on the RDC, then it was applied and programmed into ADHD-VR diagnostic tool prototype with ML application, as follows:

1. The ADHD-VR diagnostic tool prototype is a serious game that can adjunctively help professionals diagnose ADHD and uses the DSM 5 ADHD diagnosis criteria as a gold standard. This tool is intended for health-use purposes; therefore, it resulted in the game being used under supervision, thus differing from the usual daily game applications.
2. The ADHD-VR diagnosis tool prototype consisted of Indonesian local content and culture and was designed as a simulation 360-degree game.
3. The ADHD-VR diagnosis tool prototype can be used for school-aged children.
4. The concept of the ADHD-VR diagnostic tool is to immerse students in a virtual classroom. Participants can look around in every direction, interact with virtual objects (books, balls, chairs, and tables) using controllers that emulate the user’s hand, and walk anywhere as long as they are within the limits allowed by the game. To emulate the real world, the visual appearance of virtual objects and surroundings needs to be as close as possible to real objects, although the limit would be within the processing power of the VR equipment itself. The general direction for the ADHD-VR diagnostic tool was to create a local Indonesian elementary classroom, something that is familiar for the participants especially if this was their first time entering the virtual world and to give a sense of “task” for the participants as if he/she was given an instruction from the teacher. The room area is approximately 5 × 5 m, while the game area is approximately 3 × 3 m. The game area is the area where collectible objects appear.
5. In the ADHD-VR diagnostic tool, the participant’s point of view is from the eye of a virtual character (student) rendered inside the VR headset/oculus (Figure 3). This point of view is considered as “first person” since the participant sees the surroundings as if he/she is inside the classroom. While the participant freely walks around the virtual environment (virtual classroom), there are various obstacles, such as tables, chairs, and various types of walls that restrict movement. These obstacles are required for the game, not just for immersion, but also as part of the game mechanics itself. Participants can interact with limited objects in the virtual classroom. Meanwhile, tables and chairs are not movable but are static objects placed in the room as part of the virtual environment. Participants can only interact with books and balls to an extent (such as grab, throw, and place) as part of the game objectives.

6. The ADHD-VR diagnostic tool uses the Oculus Rift headset with hand controllers (Figure 3).
7. The ADHD-VR diagnostic tool was created using the Unity application, which will be converted into an a.apk file and installed into Oculus. The game data will be stored in the MongoDB database. Currently, ML application is separated from VR applications. Data from the ADHD-VR diagnostic tool is stored in the database, from the database it is processed using ML application.
8. The total duration of the ADHD-VR diagnostic tool examination is approximately 30 min, which consists of two levels of playing (tutorial and examination).
9. In this study, an ML application much more fitting for small datasets was designed with low complexity to avoid overfitting the model to the data. Thus, the study chose a simpler classifier model, such as the “decision tree,” as a predictive model for small datasets; it is easy to understand and interpret, perfect for visual representation, requires little data preprocessing, can work with numerical and categorical features, and can be used for regression (continuous real-valued output) or classification (categorical output). This study focused on the classification output based on the DSM 5 ADHD diagnostic output (hyperactivity-impulsivity type, inattentive type, combined type, and ADHD not otherwise specified). The classification and regression tree (CART) algorithm was used for classification tree construction, which is based on binary splitting of the attributes. CART uses’ Gini’ impurity index splitting measure. The CART algorithm was selected because it can handle both numerical and categorical data and multi-output problems, requires relatively little effort for data preparation, and non-linear relationships between parameters do not affect tree performance (16).
10. The ADHD-VR diagnostic tool procedure:
a) The participant is explained how to use the ADHD-VR diagnostic tool before he/she starts the examination, such as being supervised, act as a student in the classroom, tasks that need to be completed, and duration of the examination, and when they feel dizzy, tired, or have any other complaints, they can stop the examination at any time.
b) The ADHD-VR diagnostic tool examination starts when the participant turns on the Oculus Rift and the menu appears. The participants were instructed to choose the tutorial button that appeared on the oculus screen. In this part, the participant is asked to practice the hand controller unit, grab, and place the object. The participant is asked to put one object (book or ball with different sizes, colors, and pictures depending on the level of examination) into the box according to the visual instructions that appear in the classroom table. The tutorial level is completed when the participant can put the object correctly into the box at least eight times out of ten (80% correct). The tutorial level helps the participant become familiar with the Oculus Rift and hand controller unit. Although it has some learning points, it does not affect the examination results.
c) After completing the tutorial, the participant can continue to the next level (examination level) by pushing the examination level button that appears on the oculus screen.
d) At the examination level, the participant is asked to put objects into the box correctly according to instructions, such as putting three balls into the correct box, putting two balls and one book into the correct box, putting one ball and two books into the correct box, and putting three books into the correct box. They need to complete the instruction in order; if it is said to put two balls and one book into the box, it means that they need to put two balls first and later put the book into the box. There are two types of instruction at this level: visual (written on the whiteboard in front of the class) and auditory. The objects are defined as books (random size and color) and balls (random size and color). Objects may appear randomly in the virtual classroom and can be on the table or floor. The objects appear every 10 s. During the task completion, they need to remember the instruction while there are several distractions, such as auditory distractions (the sound of the door opening, the sound of children playing outside the classroom, or the sound of animals) and visual distractions like tables and chairs in the classroom. The duration of the examination level is designed for approximately 20–25 min.
e) After completing the entire level, the ADHD-VR diagnostic tool produces several data (parameters) that can be transferred into the ML application to predict the possibility of ADHD diagnosis. The parameters are:
i. The total number of correct objects that are put in order into the box.
ii. The total number of correct instructions that have been completed (in order).
iii. The total number of incorrect objects that are put in order into the box.
iv. The total number of incorrect instructions that have been completed (in order).
v. The total number of objects that have not been put into the box after the game is over.
vi. The total time duration between the instruction is given and task completion.
vii. The total duration of time to remember each instruction (both visual and auditory).
viii. The ratio between correct objects that are put into the box and the total objects that appear in the virtual classroom.
ix. The ratio between incorrect objects that are put into the box and the total objects that appear in the virtual classroom.
x. The total duration of time to put objects into the box after the instruction is given (both visual and auditory).
xi. The total number of correct objects that are put into the box before the instruction is given completely.
xii. The total number of incorrect objects that are put into the box before the instruction is completely given.

## Discussion

The development of the ADHD-VR diagnostic tool with ML followed the RDC with the DSM 5 ADHD diagnostic criteria as a gold standard supposed to adjunct the ADHD diagnosis. VR was selected as the ADHD diagnostic tool because it can produce immersive experiences and interactive activities that push more vivid images for participants. The participants were put into the virtual classroom and it created a mental feeling such as sensation and perception of “being there” (“presence”). Thus, it might trigger a certain level of likelihood of interacting and reacting, as if the participants were in the real classroom. During the feasibility testing, participants mentioned that they felt as if they were in their real classroom and the only limitation was their restricted movement in the VR classroom, but it did not reduce their “presence” sensation. These experiences are very important in ADHD-VR diagnostic tools because a higher likelihood of reality experiences may be correlated with higher diagnostic levels (2, 22, 31, 32).

In this study, we developed an ADHD-VR diagnostic tool prototype using ML applications. The tool was designed based on the RDC, which attempted to produce a simple tool with minimal distractions that could detect ADHD symptoms. For example, the child used an Oculus Rift with a hand controller. Instead of experiencing an immersive virtual classroom, children can grab, release, throw, and place objects using their virtual hands. Additionally, the virtual classroom was designed in a limited space. There are several advantages of the design, such as its ability to limit physical movements; children only need a small physical space to operate the VR digital game, which allows them to perform actions while standing or sitting (2). Furthermore, the virtual classroom was designed in a much more frivolous, calm, and simple environment; therefore, it might produce less motion sickness and help the child maintain the activity based on the instructions. Furthermore, instructions were given in two modes: visual and auditory. During task completion, objects appeared continuously and noise became distractors for the participants. Thus, this arrangement may detect problems related to selective attention, self-organization, sustained and range of attention, and impulse control skills that are usually encountered in children with ADHD. The whole arrangement and the tasks were to try to detect the inattentive, hyperactive, and impulsive symptoms objectively in a more realistic environment (VR) in children with ADHD that covered the DSM 5 diagnostic criteria for ADHD. However, further studies are required to validate the effectiveness of this treatment.

The whole design had diagnostic values such as children could grab and release objects from their surroundings and place them in the correct box within a “working space.” Based on the ConvNet theoretical background, the ADHD-VR diagnostic tool prototype contained several sequences of layers (activities) within which children could walk, grab, release, throw, and place objects that have diagnostic value. After the child grabbed/released/threw/placed an object from the toolbox, a new object of the same or different type would spawn to take its place. Thus, more than one layer of a given type could be present in the prototype. While computing, a display in front of the child reported the status of playing (23, 33). Upon completion, the ADHD-VR diagnostic tool prototype might report the accuracy of the ADHD diagnosis from the computed input data against a standardized testing set; in this way, the clinical symptoms of ADHD were based on the DSM 5 ADHD diagnostic criteria. Therefore, the input and output data from the ADHD-VR diagnostic tool were more precisely transformed into a single value for diagnostic purposes. Therefore, this tool is likely to be an objective and valuable diagnostic tool for ADHD in the coming years.

Since traditional parents’ or teachers’ ADHD behavior rating scales have subjective components, additional or more objective assessments of childhood ADHD tools have been recommended (34). Several studies have shown that VR technology has several benefits such as the feeling of enjoyment evoked by the experiences of VR itself in children. Moreover, VR technology is said to be effective and has objective components to assess ADHD behavior combined with traditional psychiatric examination and other continuous performance tests in children, because the ADHD-VR diagnostic tool has better ecological validity since children’s sustained attention is evaluated in more realistic settings (14, 19). Instead of the advantages of ADHD-VR diagnostic tool, there are also several limitations. Challenging tasks that come from visual and auditory instructions, either with/without distractors may have time delay from the moment that participants (players) capture the instructions to the task completion-feedback; this delay can be perceived as inattentive period by the ADHD-VR diagnostic tool and can influence the ML outcome. This is called the latency of the system (35). Therefore, the duration of time to remember each instruction (both visual and auditory) is being one of several others parameter in ADHD-VR diagnostic tool. The second important limitation of ADHD-VR diagnostic tool is different distance perception both in visual and auditory instructions compared to a real situation. Depending on the design of ADHD-VR diagnostic tool, hence it is essential to take into account these limits; because it can certainly modify participants’ reactions (36). Thus, the ADHD-VR diagnostic tool in this study applied consistent intonation of auditory instructions and used consistent font of visual instruction to minimize this distance perception. Since VR and ML platforms are still in the developmental stages, few studies have reported their use and effectiveness. VR and ML applications appear to be promising for clinical purposes, including diagnosis and treatment; however, further studies are necessary (18, 31, 37).

## Conclusion

In conclusion, this study developed an ADHD-VR diagnostic tool with ML based on a constructed RDC. This approach may be useful for other professionals who may also develop robust ADHD-VR or other VR diagnostic tools that are important in health services, especially mental health. Therefore, the results of this study can be used as a reference for future studies in similar contexts. The sole limitation of this study was that it did not include the effectiveness of the ADHD-VR diagnostic tool results. Thus, an effectiveness study needs to be conducted soon so that it can be applied in a clinical setting.

## Data availability statement

The original contributions presented in this study are included in the article/supplementary material, further inquiries can be directed to the corresponding author.

## Ethics statement

The studies involving human participants were reviewed and approved by the Ethics Committee of the Faculty of Medicine, University of Indonesia, Jakarta, Indonesia (reference number: KET-583/UN2.F1/ETIK/PPM.00.02/2019). Written informed consent to participate in this study was provided by the participants’ legal guardian/next of kin. Written informed consent was obtained from the individual(s) for the publication of any potentially identifiable images or data included in this article.

## Author contributions

TW contributed to the design of the manuscript, literature review, draft and preparation, and writing of the manuscript. NW contributed to the literature review and manuscript preparation. ST, RI, FK, RB, and BD conceptualized and reviewed the manuscript. All authors contributed to the article and approved the submitted version.
